# Identification of a Gene Signature to Aid Treatment Decisions by Integrated Analysis of Mutated Genes Between Primary and Metastatic Prostate Cancer

**DOI:** 10.3389/fgene.2022.877086

**Published:** 2022-04-12

**Authors:** Qinyu Li, Xueyan Xiao, Bingliang Chen, Guoda Song, Kai Zeng, Jianping Miao

**Affiliations:** ^1^ Department of Urology, Tongji Hospital, Tongji Medical College, Huazhong University of Science and Technology, Wuhan, China; ^2^ Department of Gastroenterology, Union Hospital, Tongji Medical College, Huazhong University of Science and Technology, Wuhan, China; ^3^ Department of Geriatrics, Tongji Hospital, Tongji Medical College, Huazhong University of Science and Technology, Wuhan, China

**Keywords:** metastasis, mutation, prostate cancer, treatment decision, unsupervised consensus clustering

## Abstract

Prostate cancer is one of the most common malignancies in males. Despite the recent development of advanced diagnostic platforms and treatment, patients with metastatic disease still have a poor five-year survival rate. Cancer metastasis is correlated with the characteristics of the tumor microenvironment and is significantly associated with patient prognosis. In this study, we obtained mutated genes with significant differences between primary and metastatic prostate cancer from the COSMIC database. Unsupervised consensus clustering was used based on the 1,051 genes obtained, and two PCa clusters were identified, which exhibited different prognostic outcomes and immune characteristics. Next, we generated a scoring system and evaluated the prognostic value of riskscore and its potential to aid treatment decisions in clinical practice. The riskscore could be applied to predict patients’ response to immunotherapy and sensitivity to Docetaxel. In conclusion, this study performed an integrated analysis of mutated genes between primary and metastatic prostate cancer and provides a novel assessment scheme to precisely select treatment strategies.

## Introduction

Prostate cancer (PCa) is the second most common type of cancer diagnosed in males (Sung et al., 2021). While several patients run an indolent course, most patients present with high-risk localized, locally advanced, or metastatic cancer (Teo et al., 2019). Despite localized prostate cancer exhibiting long-term survival, metastatic prostate cancer remains largely incurable even after intensive treatment (Wang et al., 2018). It has been reported that more than ninety percent of cancer-related deaths result from metastasis, and most prostate cancer patients die from metastasis (Rycaj et al., 2017). Therefore, exploring those genes with significant differences between primary and metastatic prostate cancer may help us to predict the prognosis of patients and formulate a more effective treatment regimen.

Metastasis is processed by the mechanisms by which tumor cells invade local tissues, reach the circulation, and colonize distant organs (López-Soto et al., 2017). Recent research has suggested that immune cells can regulate these steps of metastasis by influencing the extracellular matrix (ECM) (Blomberg et al., 2018). ECM remodeling can facilitate metastasis by influencing the architecture of the surrounding tissue to favor tumor cell invasion (Ghajar and Bissell, 2008) or allowing the release and diffusion of the pro-tumoral signaling molecules (Cox and Erler, 2011). Currently, the clinical successes of immunotherapy, such as immune checkpoint blockade, have revolutionized cancer therapeutics, and astonishing clinical responses have been achieved in several types of cancers (van den Bulk et al., 2018). Immunotherapy results in long-term durable remission in some advanced cancer patients (Ganesh et al., 2019). However, a large proportion of patients cannot benefit from checkpoint blockade. Therefore, how to choose suitable treatment is critical in clinical practice, and the development of immunotherapy calls for a better understanding of the influence of immune regulation on metastasis to enhance the treatment efficacy for patients with metastatic disease.

COSMIC, the Catalogue Of Somatic Mutations In Cancer, contains the most detailed and comprehensive materials of somatic mutations in human cancer (Tate et al., 2019). Its latest release includes almost 6 million coding mutations across 1.4 million tumor samples. In this study, we downloaded mutation data and corresponding sample features and performed Chi-square test to identify those genes with significant differences in mutation frequencies between primary and metastatic prostate cancer. Then, we filtered these genes by the univariate Cox regression method, performed unsupervised clustering method and identified two PCa clusters based on these mutated genes. Comprehensive analysis revealed that the two subclasses were significantly different in progression-free survival, characteristics of the immune microenvironment and the expression of immune checkpoint genes. Moreover, we extracted the feature genes to construct a riskscore by principal component analysis and evaluated the prognostic value of the riskscore and its potential to aid treatment decisions in clinical practice.

## Materials and Methods

### Data Collection and Pre-Processing

Transcriptional data (read counts), clinical characteristics and somatic mutation data were acquired from the TCGA database (Supplementary Table S1). Next, we downloaded four datasets with the same platform (Affymetrix GPL570) from the GEO database: GSE69223 (Meller et al., 2016) (N = 30), GSE32448 (Derosa et al., 2012) (N = 80), GSE55945 (Arredouani et al., 2009) (N = 21), and GSE46602 (Mortensen et al., 2015) (N = 50). Thereafter, we adjusted the background by using the “RMA” algorithm of the “affy” R package (Gautier et al., 2004) and removed the batch effect by the “ComBat” algorithm of the “sva” package (Leek et al., 2012). Therefore, we can merge the four datasets as the validation cohort. Moreover, GSE21034 (Taylor et al., 2010) (N = 370) was utilized to validate the prognostic value.

### Identification of Mutated Genes Between Primary and Metastatic Prostate Cancer

COSMIC is currently the broadest database of mutations in human cancer (Forbes et al., 2017). COSMIC mutation data (Genome Screens) and corresponding sample features were downloaded, and we estimated the frequency of each mutation site. Chi-square test was applied to discover the mutated genes with significant differences between primary and metastatic prostate cancer. Next, we executed prognostic analysis for each gene discovered by univariate Cox regression, and the genes related to prognosis with *p*-value < 0.05 were extracted for further analysis.

### Identification of PCa Subclasses

Unsupervised consensus clustering of the obtained genes was executed by using the k-means algorithm in the “ConsensusClusterPlus” package (Wilkerson and Hayes, 2010), which was repeated 1,000 times to ensure the stability of the classification. Survival differences between the two clusters were visualized by Kaplan–Meier curves. To explore the molecular characteristics of the two clusters. The “c2.cp.kegg.v7.4.symbols” gene set was downloaded from the MSigDB database, and we applied the “GSVA” package (Hänzelmann et al., 2013) to perform the GSVA analysis.

### Immune Infiltration Levels Between PCa Subclasses

The “ssGSEA” method was performed to estimate the infiltration degrees of 28 immune cells by using the “GSVA” package. Estimate is commonly used to calculate scores reflecting the infiltration levels of immune cells and stromal cells in the tumor microenvironment by the package “estimate” (Yoshihara et al., 2013). We applied the estimate algorithm to calculate the ImmuneScore and StromalScore of each sample. Additionally, the correlation between the expression of immune checkpoint genes and androgen receptor between the two clusters was estimated.

### Generation of Riskscore

The Pearson correlation coefficients of mutated genes with the identified PCa subclasses were estimated, and the signature genes A and B were obtained based on the correlation coefficients. Then, we applied the Boruta algorithm to the positively and negatively correlated genes to select feather genes. Finally, principal component analysis (PCA) was performed to estimate the first principal components of signature genes A and B. We defined the riskscore of each sample as follows:

Riskscore=∑PC1B−∑PC1A

### Correlation Between Clinical Parameters, Immune Infiltration and Riskscore

The difference in the riskscore in patients stratified by clinical parameters was estimated to expound the effect of the riskscore on cancer progression. Moreover, immune cell infiltration, ImmuneScore, StromalScore and the expression of immune checkpoint genes were also assessed between the high- and low-risk groups.

### Tumor Mutation Burden Analysis

Tumor mutation burden (TMB) has been demonstrated as a predictive biomarker to identify whether patients with cancer can respond to immune checkpoint inhibitors well (Merino et al., 2020). Here, we explored the correlation between TMB and riskscore. Furthermore, we divided patients into four subgroups based on the median value of riskscore and TMB. Survival differences of the four subgroups were visualized by Kaplan–Meier curves.

### Benefits of the Riskscore to Aid Treatment Decisions

Since the comparison of the expression of different immune checkpoint genes between the high- and low-risk groups was performed, here we used an immunotherapeutic cohort (IMvigor210 cohort) as a validation cohort (Mariathasan et al., 2018). We first evaluated the influence of the riskscore on the prognosis of patients treated with immunotherapy. Then, the riskscore of patients with different clinical statuses after treatment were compared. Finally, transcriptional data of tumor cell lines and IC50 values of antitumor drugs from the GDSC database were used to perform the drug sensitivity analysis by using the “pRRophetic” package (Geeleher et al., 2014).

### Statistical Analysis

All analyses were performed in RStudio 4.0.4. Correlation analysis was computed by the Spearman method. Student’s *t* test and the Wilcoxon test were applied for two-group comparisons. Correspondingly, the Kruskal–Wallis test and one-way ANOVA were used for multiple groups. Statistical significance was defined as *p*-value < 0.05.

## Results

### Genetic Alterations in Prostate Cancer

**FIGURE 1:**
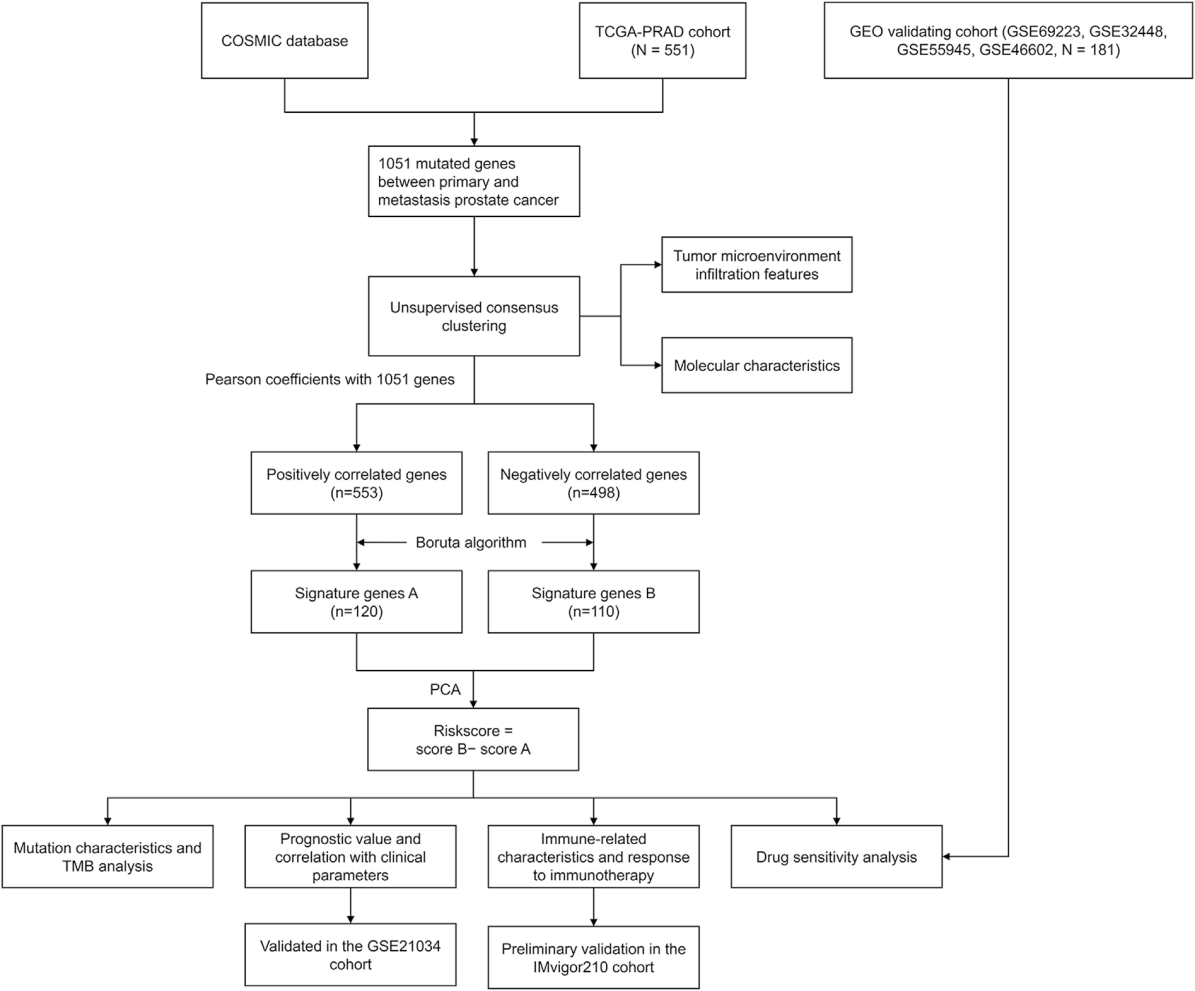
Flow chart of this study.

**FIGURE 2:**
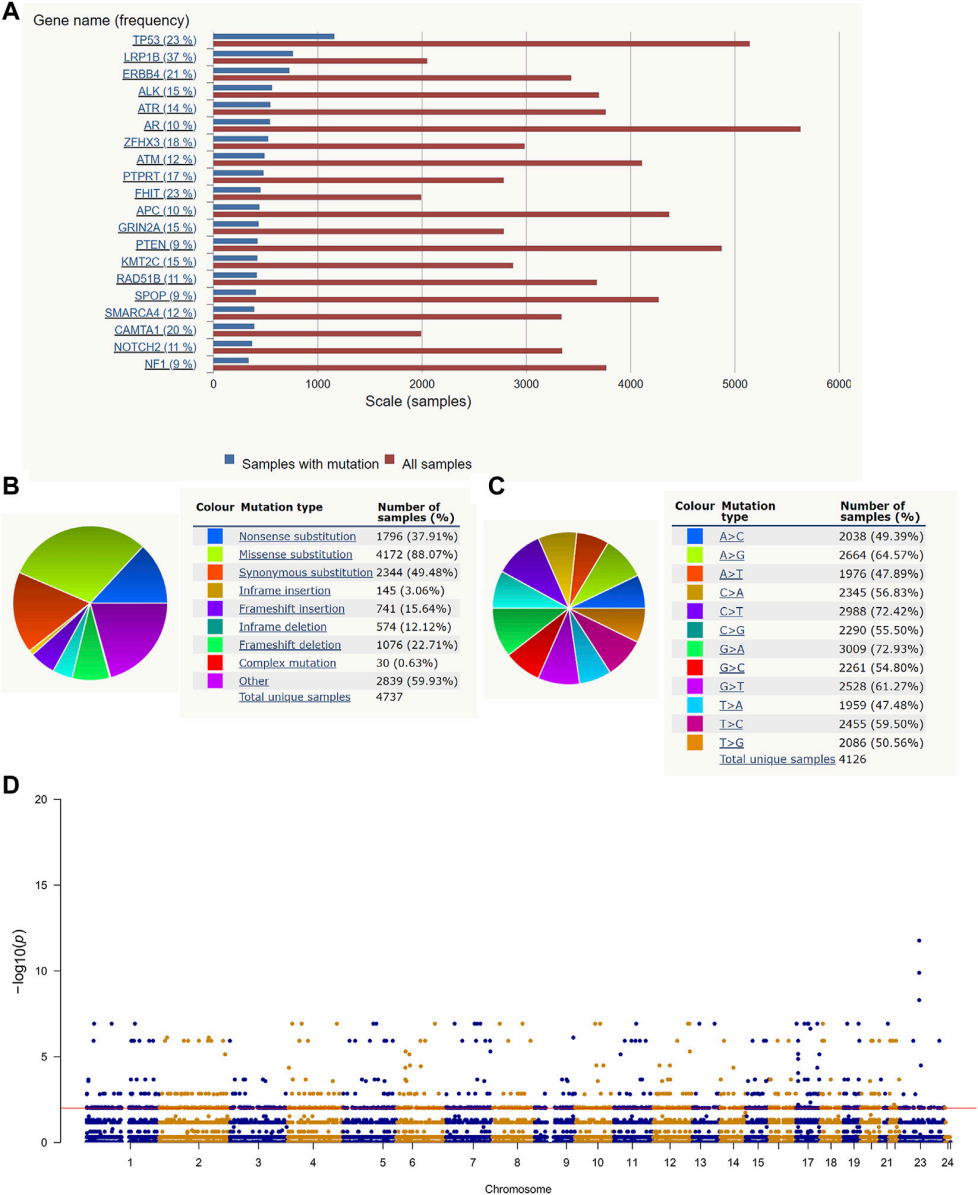
Gene mutation in prostate cancer: **(A)** The top 20 mutated genes in prostate cancer. **(B,C)** The distribution of different types of mutations in prostate cancer. **(D)** The Manhattan plot of mutation sites that have significantly different mutation frequencies between primary and metastatic prostate cancer.

The roadmap of this study is illustrated in Figure 1. The top 20 mutated genes for prostate cancer are shown in Figure 2A, and the mutation frequencies are displayed next to the gene name. Furthermore, Figures 2B,C shows the distribution of different types of mutations for prostate cancer. Missense substitution (88.07%), synonymous substitution (49.48%) and nonsense substitution (37.91%) were the main types of mutations, and the substitution mutations mainly included G > A (72.93%), C > T (72.42%), A > G (64.57%), and G > T (61.27%). Moreover, the Manhattan plot depicted mutation sites that had significant differences between primary and metastatic prostate cancer (Figure 2D).

### Identification of PCa Subclasses

We obtained 3,716 mutated genes with significant differences between primary and metastatic prostate cancer by the Chi-square test (Supplementary Table S2). Thereafter, we explored the prognostic value of these genes for progression-free survival (PFS) by the univariate Cox method, and 1,051 genes were extracted (Supplementary Table S3) for further analysis.

**FIGURE 3:**
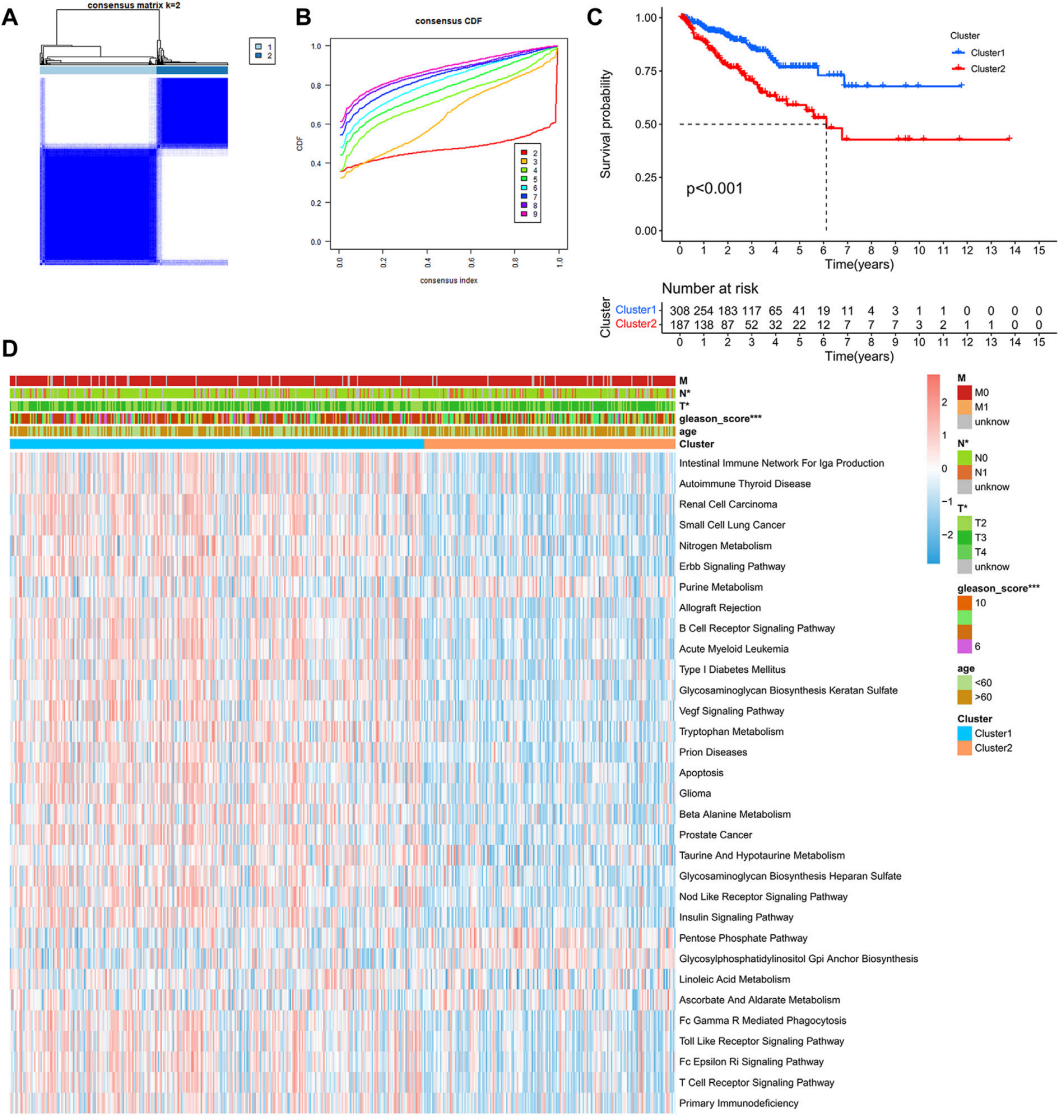
Identification of PCa subclasses by unsupervised consensus clustering: **(A)** Matrix heatmap of the K-means clustering using 1,051 mutated genes between primary and metastatic prostate cancer. **(B)** CDF curve of the clustering result. **(C)** Kaplan–Meier survival curve of PFS between different clusters. **(D)** Heatmap of GSVA enrichment based on KEGG pathways between different clusters. (*p < 0.05, **p < 0.01, ***p < 0.001).

After comprehensive consideration of CDF curves and delta area, we chose *k* = 2 as the optimal cluster number for the clustering (Figure 3). Finally, 308 patients were assigned to Cluster 1, and 187 patients were assigned to Cluster 2. We also applied t-SNE dimension reduction, and the results suggested that the discrimination among subgroups was decent (Supplementary Figure S1). Survival curves suggested that Cluster 2 had a significant survival advantage compared with Cluster 1 (Figure 3C). Moreover, GSVA analysis and limma analysis (log FC > 0.2, adjusted *p*-value < 0.05) were performed. Significant differences in pathways related to cancer progression, such as the ERBB signaling pathway and VEGF signaling pathway, and pathways associated with the immune response, such as the B cell receptor signaling pathway and T cell receptor signaling pathway, were observed between the two clusters (Figure 3D).

**FIGURE 4:**
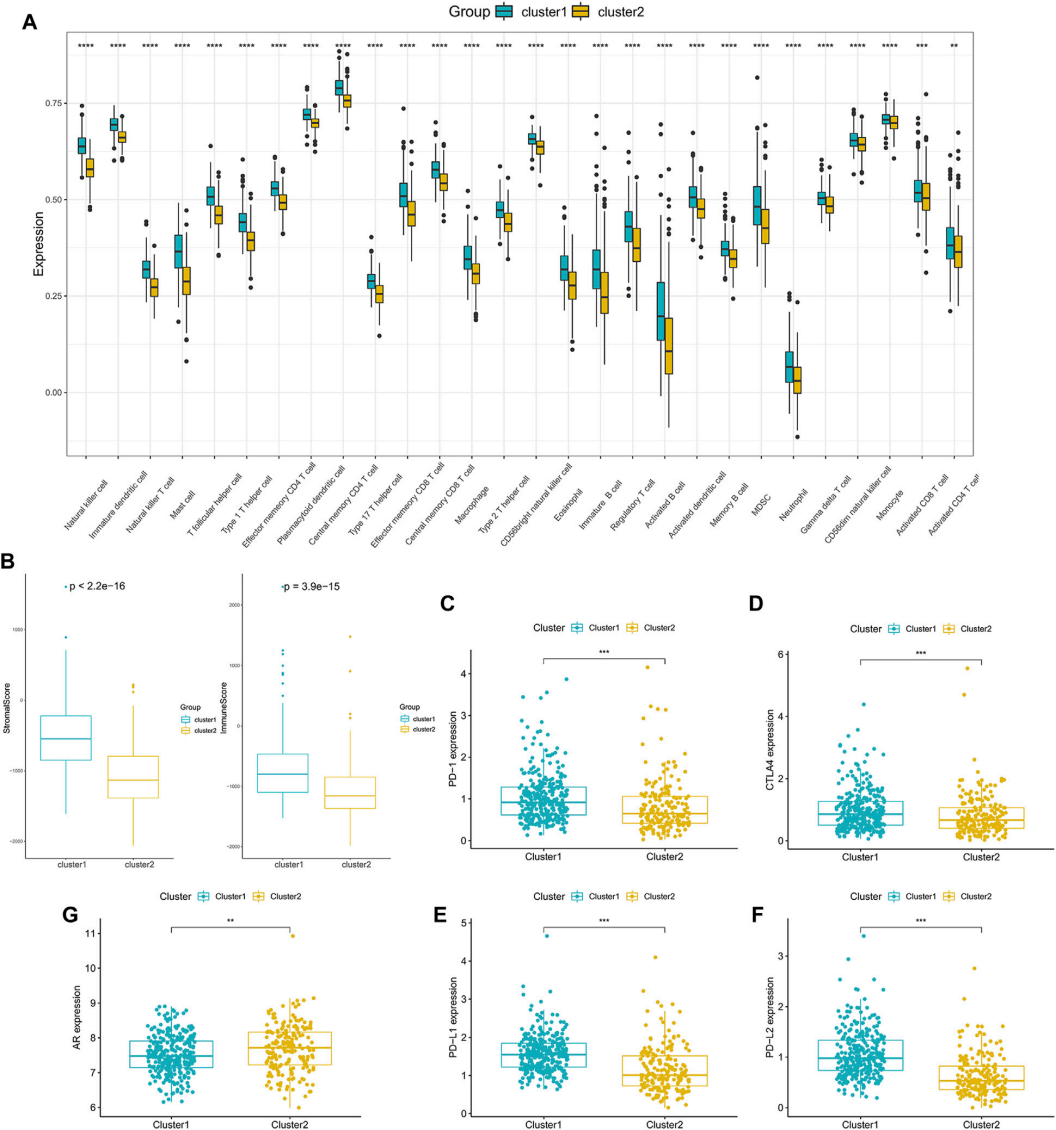
Tumor microenvironment characteristics of different PCa subclasses: **(A)** The proportions of TME cells in the two clusters. **(B)** ImmuneScore and StromalScore of the two subgroups. **(C–F)** The expression of the immune checkpoint genes PD-1 **(C)**, CTLA4 **(D)**, PD-L1 **(E)** and PD-L2 **(F)** between the two clusters. **(G)** The expression of AR in the two clusters. (*p < 0.05, **p < 0.01, ***p < 0.001, ****p < 0.0001).

In order to delve into the immune-related characteristics of the two clusters, the infiltration levels of immune cells were compared between the two clusters. A significant difference was observed in the infiltration degree of all immune cells, and all immune cells infiltration were lower in Cluster 2 (Figure 4A). Moreover, the results indicated that both the StromalScore and ImmuneScore of Cluster 2 were significantly lower compared with Cluster 1 (Figure 4B). What’s more, the expression of immune checkpoint genes, including CTLA4, PD-1, PD-L1 and PD-L2, appeared to be decreased in Cluster 2 (Figures 4C–F). However, the expression of AR was higher in Cluster 2 than in Cluster 1 (Figure 4G), which is consistent with the poor survival of Cluster 2.

### Construction of the Riskscore For Each Sample and the Prognostic Value

**FIGURE 5:**
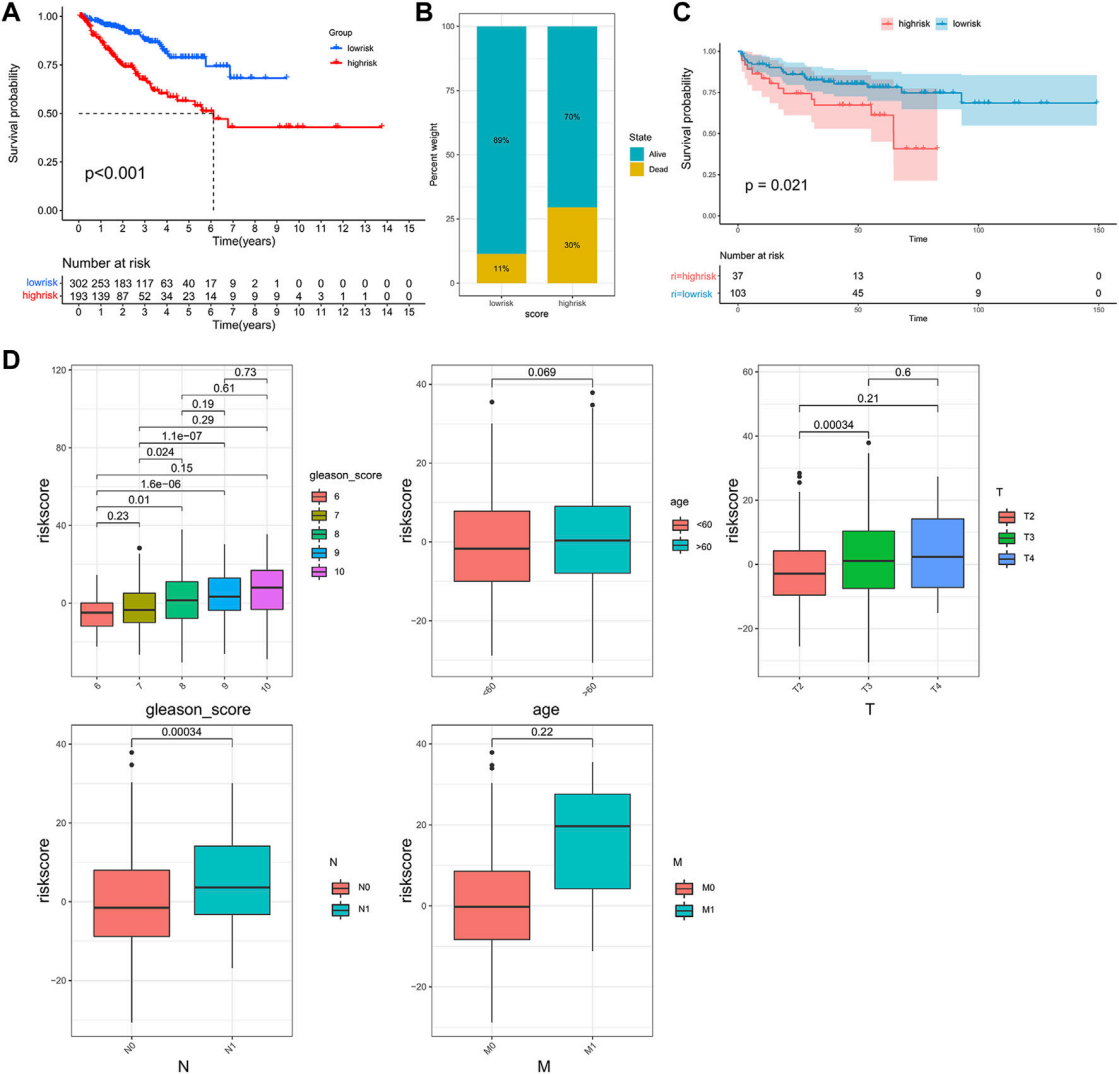
Construction of the riskscore and prognosis analysis. **(A)** Kaplan–Meier survival curve between high- and low-risk subgroups in the TCGA cohort. **(B)** The proportion of the survival rate between the high- and low-risk subgroups. **(C)** Kaplan–Meier survival curve between the high- and low-risk subgroups in the validation cohort, GSE21034. **(D)** The differences in riskscore between patients with different clinicopathological parameters (Gleason score, age, T stage, N stage, M stage).

Previous results indicated that the subclass was closely associated with the prognosis and immune infiltration levels of patients. However, this population-based classification cannot be directly used in clinical practice. Therefore, we constructed a scoring system to estimate the riskscore to predict the outcome of the patients and aid in making treatment decisions. After performing the Boruta algorithm, 230 genes that were positively and negatively correlated to the subclass were defined as signature genes A and B (Supplementary Table S4). The riskscore was acquired by conducting PCA on each signature gene (Supplementary Table S5). Patients were classified into high- and low-risk groups according to the cut-off point gained by the “survminer” package (Supplementary Figure S2). The results of survival analysis showed that patients in the high-risk group had lower PFS than those in the low-risk group (Figure 5A). As shown in Figure 5B, the high-risk group possessed a higher proportion of death. In the validation cohort GSE21034, patients with higher riskscore also showed a significantly shorter median PFS (Figure 5C). Moreover, it was observed that the riskscore was elevated in the high-risk clinical group with the progression of tumor (Figure 5D), and patients who achieved complete response after treatment had a significantly lower riskscore than other outcomes (Supplementary Figure S3).

### Relationship Between Riskscore and TMB

**FIGURE 6:**
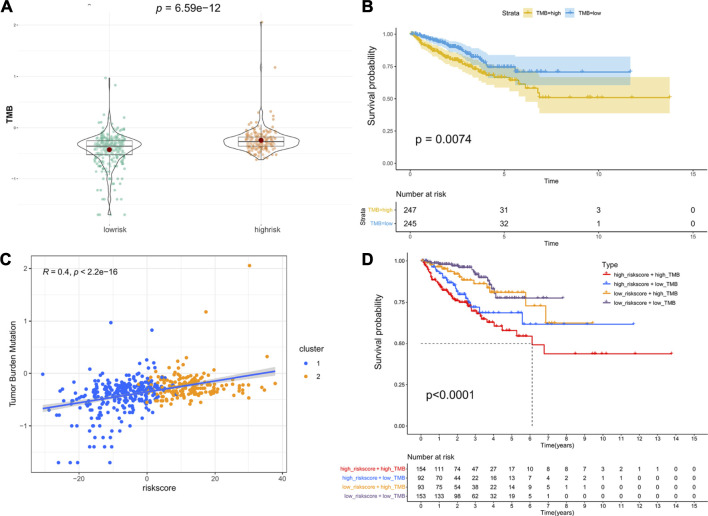
Association between riskscore and TMB: **(A)** The difference in TMB between the high- and low-risk groups. **(B)** Kaplan–Meier survival curves of PFS between the high- and low-TMB groups. **(C)** The correlation of riskscore and TMB. **(D)** Kaplan–Meier survival curves of PFS among the four subgroups stratified by riskscore and TMB.

TMB is emerging as a potential biomarker to predict the patients response to immune checkpoint inhibitors (Chan et al., 2019). Here, we evaluated the association between the riskscore and TMB. As shown in Figure 6A, patients in the high-risk group had a higher TMB than those in the low-risk group, and patients with a higher TMB had lower PFS (Figure 6B). Moreover, the correlation analysis indicated that the riskscore was positively associated with TMB (Figure 6C). Next, we combined the riskscore and TMB to divide patients into four subgroups. The patients with a high riskscore and high TMB had the shortest median PFS, and patients with a low riskscore and low TMB performed the best prognosis (Figure 6D). Finally, the mutation status of genes with high mutation frequencies in the high- and low-risk groups was visualized (Supplementary Figure S4).

### Correlation Between Immunotherapy Reactivity, Drug Sensitivity and Riskscore

**FIGURE 7:**
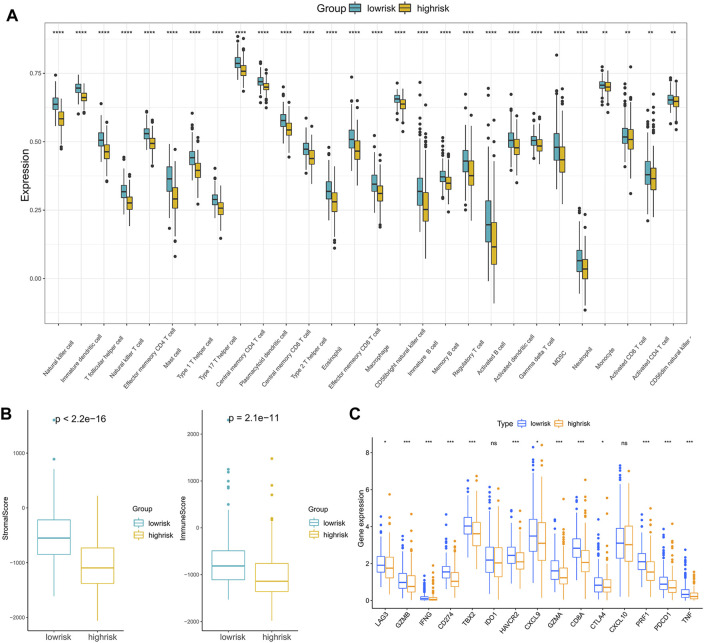
Analysis of the correlation between the riskscore and immune cell infiltration: **(A)** The immune cell infiltration levels between the high- and low-risk subgroups. **(B)** ImmuneScore and StromalScore between the high- and low-risk subgroups. **(C)** The expression of immune checkpoint genes in the high- and low-risk subgroups. (*p < 0.05, **p < 0.01, ***p < 0.001, ****p < 0.0001).

Anticancer immunotherapies involving immune checkpoint inhibitors have emerged as new therapeutic regimens (O’Donnell et al., 2019). The tumor microenvironment (TME) was proven to be tightly linked to tumor progression and metastasis (Brassart-Pasco et al., 2020) and can blunt the therapeutic response, thus affecting the clinical outcome (Wu and Dai, 2017). To further explore the correlations between patients’ response to immunotherapy and riskscore, we first compared the immune cell infiltration levels between high- and low-risk groups, and the results indicated that compared with the low-risk patients, the infiltration levels of 28 immune cells in the high-risk patients were significantly downregulated (Figure 7A).

**FIGURE 8:**
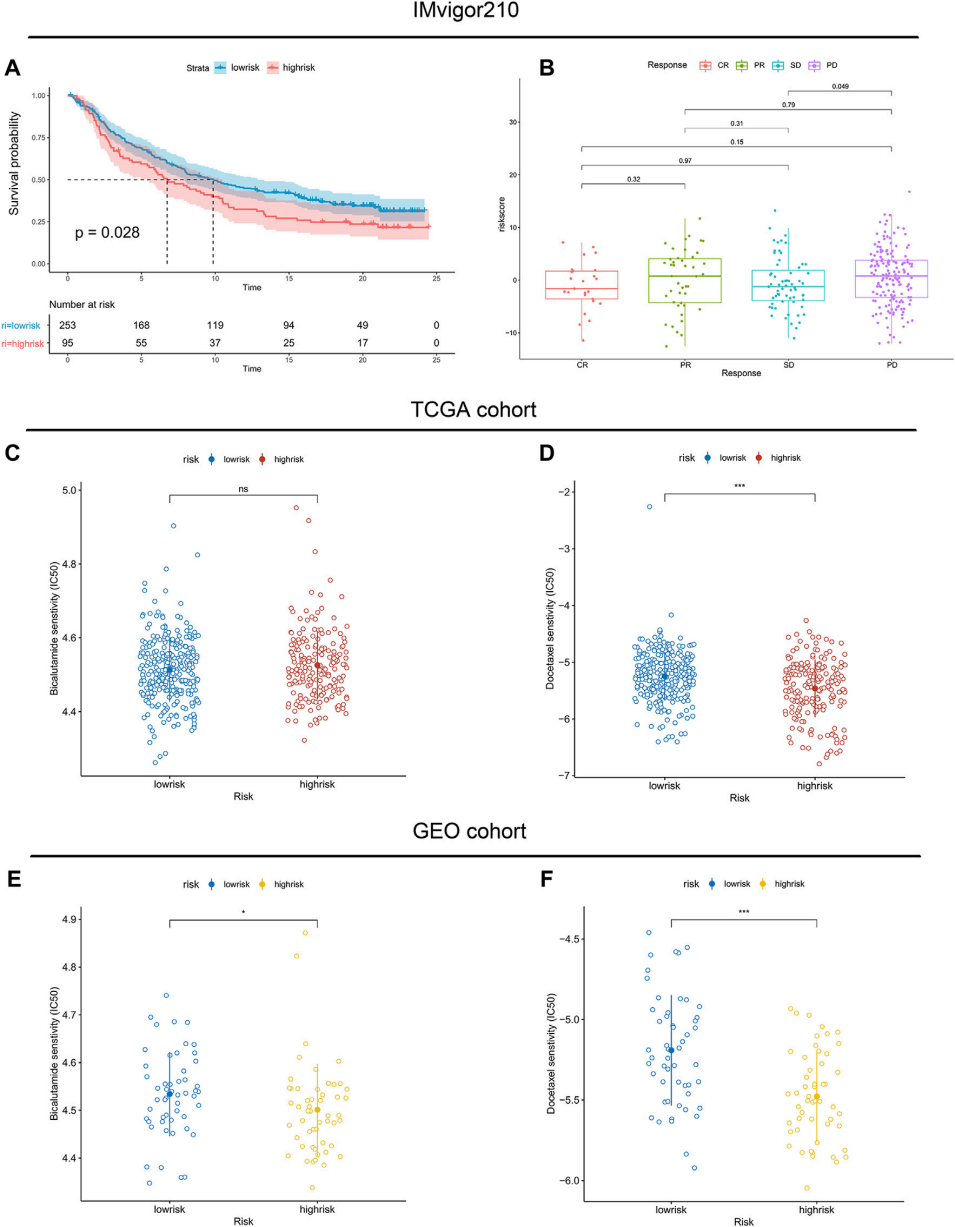
Association between immunotherapy reactivity, drug sensitivity and riskscore. **(A)** Kaplan–Meier survival curve between high- and low-risk subgroups in the IMvigor210 cohort. **(B)** The difference in riskscore among patients with different responses to immunotherapy. **(C,E)** The predicted IC50 values of Bicalutamide in the TCGA cohort and validated GEO cohort. **(D,F)** The predicted IC50 values of Docetaxel in the TCGA cohort and validated GEO cohort.

Moreover, infiltration estimation for TCGA was downloaded from TIMER 2.0 (Li et al., 2020), which includes several methods, such as XCELL, TIMER, QUANTISEQ, MCPCOUNTER, EPIC, and CIBERSORT. We correlated the immune cell infiltration levels and the riskscore, and the results also suggested that the riskscore was negatively related to most of the immune cells (Supplementary Figure S5). Additionally, the StromalScore and ImmuneScore of the high-risk patients were significantly lower than low-risk patients as well (Figure 7B). Furthermore, the associations between immune checkpoint inhibitor genes and the riskscore were evaluated. The expression of ICI genes, such as CTLA4, PD-1, PD-L1 and LAG3, were downregulated in the high-risk groups compared with the low-risk groups (Figure 7C). Finally, we validated the performance of the riskscore in the IMvigor210 cohort. As shown in Figure 8A, the low-risk patients still showed a significant survival advantage compared with high-risk patients. We evaluated the differences in riskscore among patients with different responses to immunotherapy. Patients with complete response (CR) had the lowest riskscore while patients performed progressive disease (PD) had the highest riskscore (Figure 8B). These results suggested that patients in the high-risk group may not have a satisfying response to immunotherapy.

Considering that androgen deprivation therapy and chemotherapy still play vital roles in the treatment of prostate cancer. We used the GDSC database to explore the association between the riskscore and drug sensitivity. The results revealed little difference in the predicted IC50 of Bicalutamide between the high- and low-risk groups (Figures 8C,E). However, both in the TCGA and GEO cohorts, significant differences were observed in the predicted IC50 of Docetaxel between the high- and low-risk groups (Figures 8D,F). The IC50 of Docetaxel was significantly lower in the high-risk group, suggesting that these patients are sensitive to Docetaxel.

## Discussion

Prostate cancer affects millions of men all over the world, and accounts for 7% of newly diagnosed cancers in men worldwide (Rebello et al., 2021). While the prognosis of localized PCa has a good 5-years survival rate, the 5-years survival rate of metastatic PCa decreases significantly to only 30% (Siegel et al., 2020). It is well-known that the growth and progression of prostate cancer are significantly influenced by androgen, and androgen deprivation is an effective treatment strategy which is widely used in clinical practice (Marques et al., 2005). However, among patients with metastatic disease, a substantial proportion will develop metastatic castration-resistant prostate cancer (mCRPC), which is not sensitive to androgen deprivation therapy. Therefore, the long-term prognosis for patients with mCRPC is extremely poor (Henríquez et al., 2021). On that account, it is critical to unearth the mechanism of metastasis of prostate cancer, which may assist us in predicting the prognosis of patients and forming a desirable therapeutic regimen. Although multitudinous studies have explored the correlation of some specific genes in tumor metastasis, few studies have focused on the overall mutated genes between primary and metastatic prostate cancer. Therefore, we used the COSMIC database, which contains the most detailed resource of somatic mutations in human cancer, and executed the Chi-square test to obtain mutated genes with significant differences between primary and metastatic prostate cancer.

In this study, unsupervised clustering method was conducted, and two subclasses of PCa were obtained. Then, we comprehensively assessed the two clusters of prostate cancer and explored their biological characteristics. We observed that significant differences existed between the two clusters in some carcinogenic activation signaling pathways, such as the ErbB signaling pathway (Wang, 2017) and Vegf signaling pathway (Apte et al., 2019), and pathways associated with the immune response, such as the B cell receptor signaling pathway and T cell receptor signaling pathway. Moreover, the results indicated that all the immune cell infiltration levels and the expression of immune checkpoint genes were lower in Cluster 2, which was associated with poorer survival. We supposed that the poor prognosis of patients in Cluster 2 was due to tumor immune escape.

Next, we evaluated the riskscore of each patient by using the “Boruta” algorithm and PCA analysis. Its prognostic value was demonstrated both in TCGA and GEO cohorts. Since cancer develops as a result of somatic mutation and clonal selection (Martincorena et al., 2017), herein, we correlated riskscore and TMB and found a significant positive correlation between riskscore and TMB. Moreover, we assessed the mutation status of genes with high mutation frequencies in the high- and low-risk groups. It was observed that the high-risk groups contained more mutated samples and more mutation types.

Androgen deprivation therapy is a standard treatment used in all stages of recurrent prostate cancer. However, patients will develop CRPC eventually (Gamat and McNeel, 2017). In the past, the consensus was that immunotherapy might be ineffective in prostate cancer due to the immunosuppressive microenvironment (Chakravarty et al., 2020). However, with the recent development of advanced molecular diagnostic platforms, immunotherapy has revolutionized the treatment of prostate cancer and is re-emerging as a practicable option for patients, especially for CRPC (Cha et al., 2020). Nevertheless, a key challenge for immunotherapies is that these treatments have serious adverse effects, including autoimmunity and nonspecific inflammation (Riley et al., 2019). Additionally, many patients appear to have innate or acquired resistance to immunotherapies (O’Donnell et al., 2019). Therefore, it is critical to find reliable validated biomarkers to predict the immunotherapy responsiveness of patients. In fact, it is obvious that using a single biomarker to predict benefit from immunotherapy strategies is unstable. Consequently, we extracted 230 feature genes to construct the riskscore. According to the results, all immune cell infiltration levels were higher in the low-risk groups, and immune checkpoint genes used in immune checkpoint blockade therapy, such as PD-1, CTLA4 and PD-L1, were also more highly expressed in the low-risk groups. Therefore, we suppose that patients identified as having a low riskscore may benefit from the therapeutic strategy combining immune checkpoint blockade therapy, while patients are diagnosed with a high riskscore. Since open-access data of prostate cancer cohorts accepting immunotherapy are rare, we used patients in the IMvigor210 cohort for preliminary validation. We observed that patients who reacted as complete response had the lowest riskscore while patients performed progressive disease had the highest riskscore, which is consistent with the trends of expression of immune checkpoints.

Considering it is unrealistic that utilizing immunotherapy alone can dramatically change the outcome of prostate cancer right now, the combination of conventional cytotoxic agents, androgen deprivation therapy and personalized immunotherapy is more appropriate for patients. We used the GDSC database, which is the largest public resource for information on drug sensitivity in cancer cells (Yang et al., 2013), to predict the IC50 values of drugs for treating prostate cancer. We observed that the high-risk group was more sensitive to Docetaxel than the low-risk group. In fact, Docetaxel was the first systemic therapy to demonstrate survival benefit in mCRPC and became the standard of care for mCRPC in 2004 (Teo et al., 2019). It is still recommended as a first-line treatment for mCRPC in the latest EAU guidelines (Cornford et al., 2021). Therefore, it seems reasonable that patients identified with high riskscores had a higher sensitivity to Docetaxel. Bicalutamide is a competitive androgen receptor antagonist that leads to prostate cell apoptosis and the inhibition of prostate cancer growth (Wellington and Keam, 2006). We also evaluated the IC50 values of Bicalutamide in the high- and low-risk groups. However, there were few differences in the predicted IC50 of Bicalutamide between the high- and low-risk groups. Regrettably, limited by the data currently available in the GDSC database, we could not evaluate the IC50 values of Abiraterone and Enzalutamide in this study. As second-generation androgen receptor inhibitors, they have already been recommended by the latest EAU guidelines (Cornford et al., 2021). Numerous studies have demonstrated that the second-generation androgen receptor inhibitor is associated with improved outcomes compared with bicalutamide in CRPC (Penson et al., 2016; Naiki et al., 2021; Ueda et al., 2021; Vaishampayan et al., 2021). Therefore, the drug sensitivity of Abiraterone and Enzalutamide between high- and low-risk groups needs to be further explored. Moreover, due to the lack of available immunotherapy cohorts of prostate cancer, preliminary validation was performed in the IMvigor210 cohort for bladder cancer. The ability of the riskscore to predict the immunotherapy response of patients still needs further validation in immunotherapy cohorts of prostate cancer.

## Conclusion

In summary, we selected mutated genes with significant differences between primary and metastatic prostate cancer from the COSMIC database and identified two PCa clusters that exhibited different prognostic outcomes and immune characteristics. For a better application in clinical practice, we constructed a scoring system and evaluated the prognostic value of the riskscore and its potential to aid treatment decisions. The riskscore could be applied to predict patients’ response to immunotherapy and sensitivity to Docetaxel. The results suggested that immunotherapy may benefit patients in the low-risk group, while Docetaxel is more effective for patients identified in the high-risk group.

## Website

TCGA database: https://portal.gdc.cancer.gov/; GEO database: https://www.ncbi.nlm.nih.gov/geo/; COSMIC database: https://cancer.sanger.ac.uk/cosmic; MSigDB database: https://www.gsea-msigdb.org/gsea/msigdb/; GDSC database: https://www.cancerrxgene.org/.

## Data Availability

The original contributions presented in the study are included in the article/Supplementary Material, further inquiries can be directed to the corresponding author.

## Abbreviations

COSMIC, The Catalogue of Somatic Mutations in Cancer; ECM, Extracellular matrix; FC, Fold change; GDSC, Genomics of Drug Sensitivity in Cancer; GEO, Gene Expression Omnibus; GSVA, Gene set variation analysis; PCa, Prostate cancer; PCA, principal components analysis; ROC, Receiver operating characteristic; ssGSEA, Single sample gene set enrichment analysis; TCGA, The Cancer Genome Atlas; TMB, Tumor mutation burden; TME, Tumor microenvironment.
