# Supplementary material for: Identification of a Gene Signature to Aid Treatment Decisions by Integrated Analysis of Mutated Genes Between Primary and Metastatic Prostate Cancer
Source: Front Genet. 2022 Apr 12;13:877086. doi: 10.3389/fgene.2022.877086 (PMC9041415; doi:10.3389/fgene.2022.877086)
Supplement: Supplementary file 1 [file Table1.DOCX]

Dear editorial board of the Frontiers in Genetics,

We are submitting a manuscript entitled “Identification of a gene signature to aid treatment decisions by integrated analysis of mutated genes between primary and metastatic prostate cancer” to the Frontiers in Genetics for your kind consideration of its suitability for publication. All authors have read and approved the manuscript. The material has not been submitted for publication elsewhere while under consideration for Frontiers in Genetics. There are no conflicts of interests to declare.

Prostate cancer is one of the most common malignancies in males. Despite the recent development of advanced diagnostic platforms and treatment, patients with metastatic disease still have a poor five-year survival rate. Cancer metastasis is correlated with the characteristics of the tumor microenvironment and is significantly associated with patient prognosis. In this study, we obtained mutated genes with significant differences between primary and metastatic prostate cancer from the COSMIC database. Unsupervised consensus clustering was used based on the 1051 genes obtained, and two PCa clusters were identified, which exhibited different prognostic outcomes and immune characteristics. Next, we generated a scoring system and evaluated the prognostic value of riskscore and its potential to aid treatment decisions in clinical practice. The riskscore could be applied to predict patients’ response to immunotherapy and sensitivity to Docetaxel. In conclusion, this study performed an integrated analysis of mutated genes between primary and metastatic prostate cancer and provides a novel assessment scheme to precisely select treatment strategies.

We believe that our findings could be of great interest to the readers of the Frontiers in Genetics, we do hope your favorable consideration for publication.

Sincerely,

Jianping Miao on behalf of all authors.

02.16,2022

Corresponding Author: Jianping Miao,

miaojianping888@hotmail.com

Department of Geriatrics, Tongji Hospital, Tongji Medical, College, Huazhong University of Science and Technology, Wuhan, China.
