# Supplementary material for: Identification of a Gene Signature to Aid Treatment Decisions by Integrated Analysis of Mutated Genes Between Primary and Metastatic Prostate Cancer
Source: Front Genet. 2022 Apr 12;13:877086. doi: 10.3389/fgene.2022.877086 (PMC9041415; doi:10.3389/fgene.2022.877086)
Supplement: Supplementary file 2 [file Presentation1.PPTX]

## Slide 1
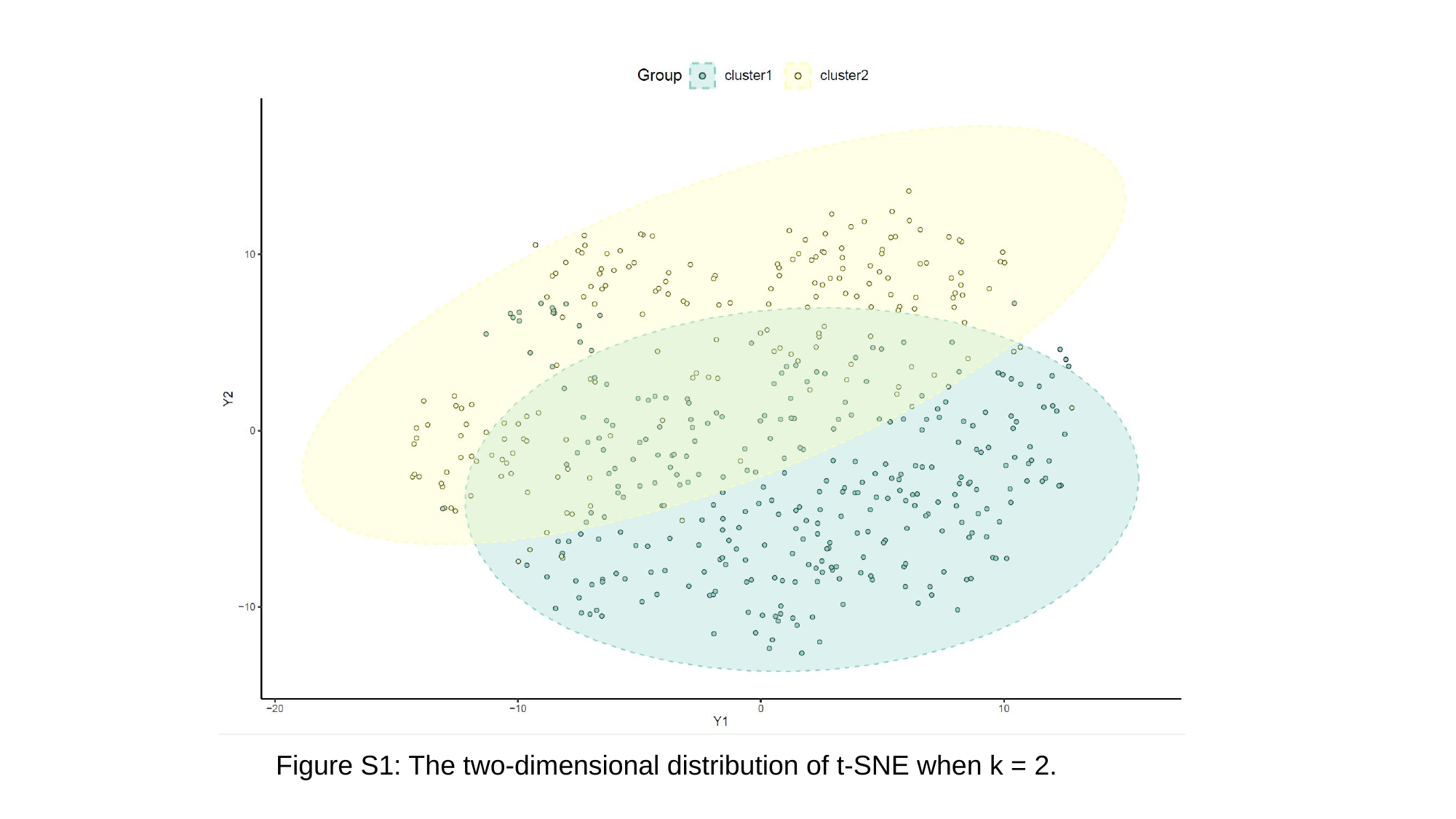

Figure S1: The two-dimensional distribution of t-SNE when k = 2.

## Slide 2
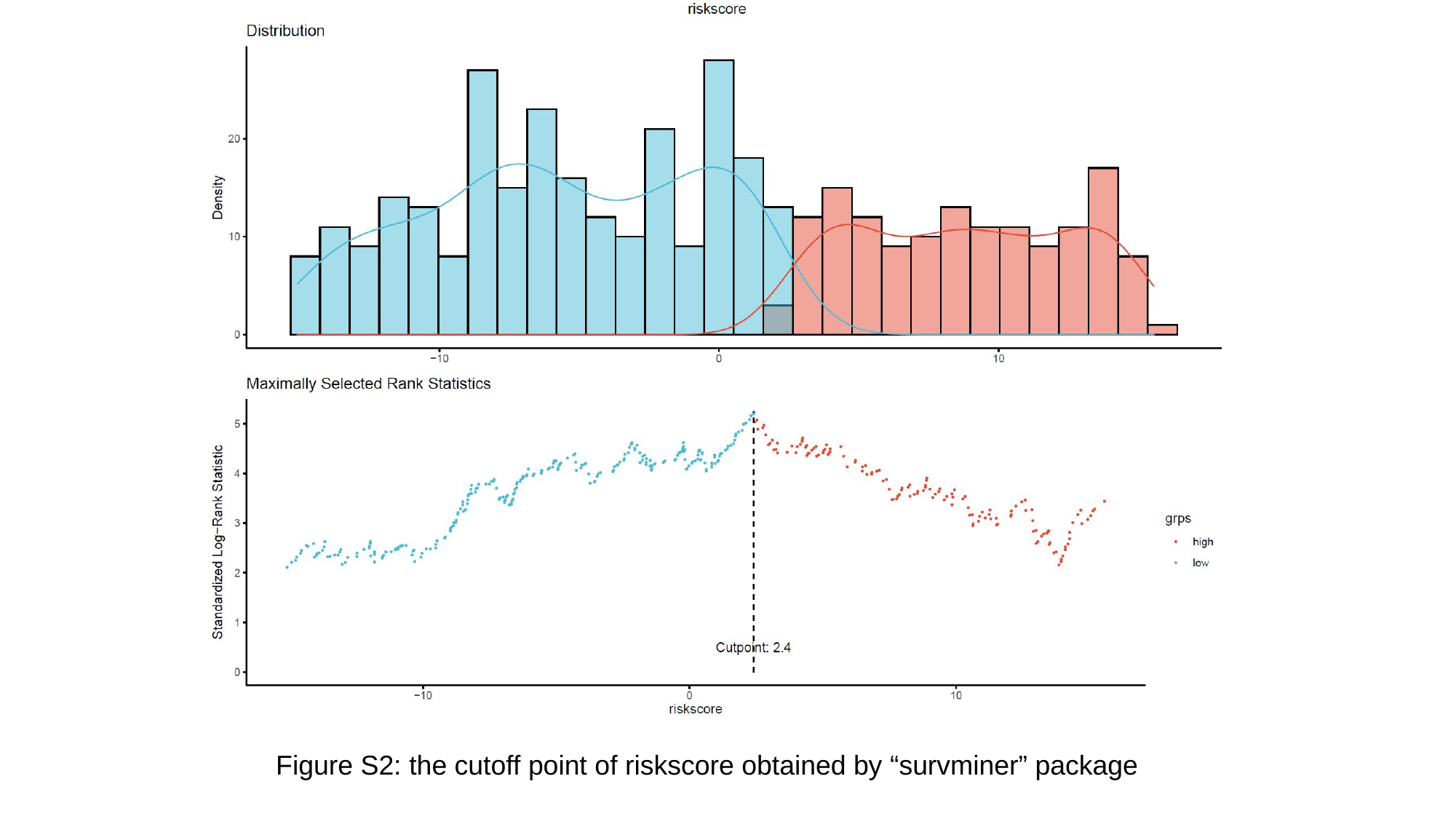

Figure S2: the cutoff point of riskscore obtained by “survminer” package

## Slide 3
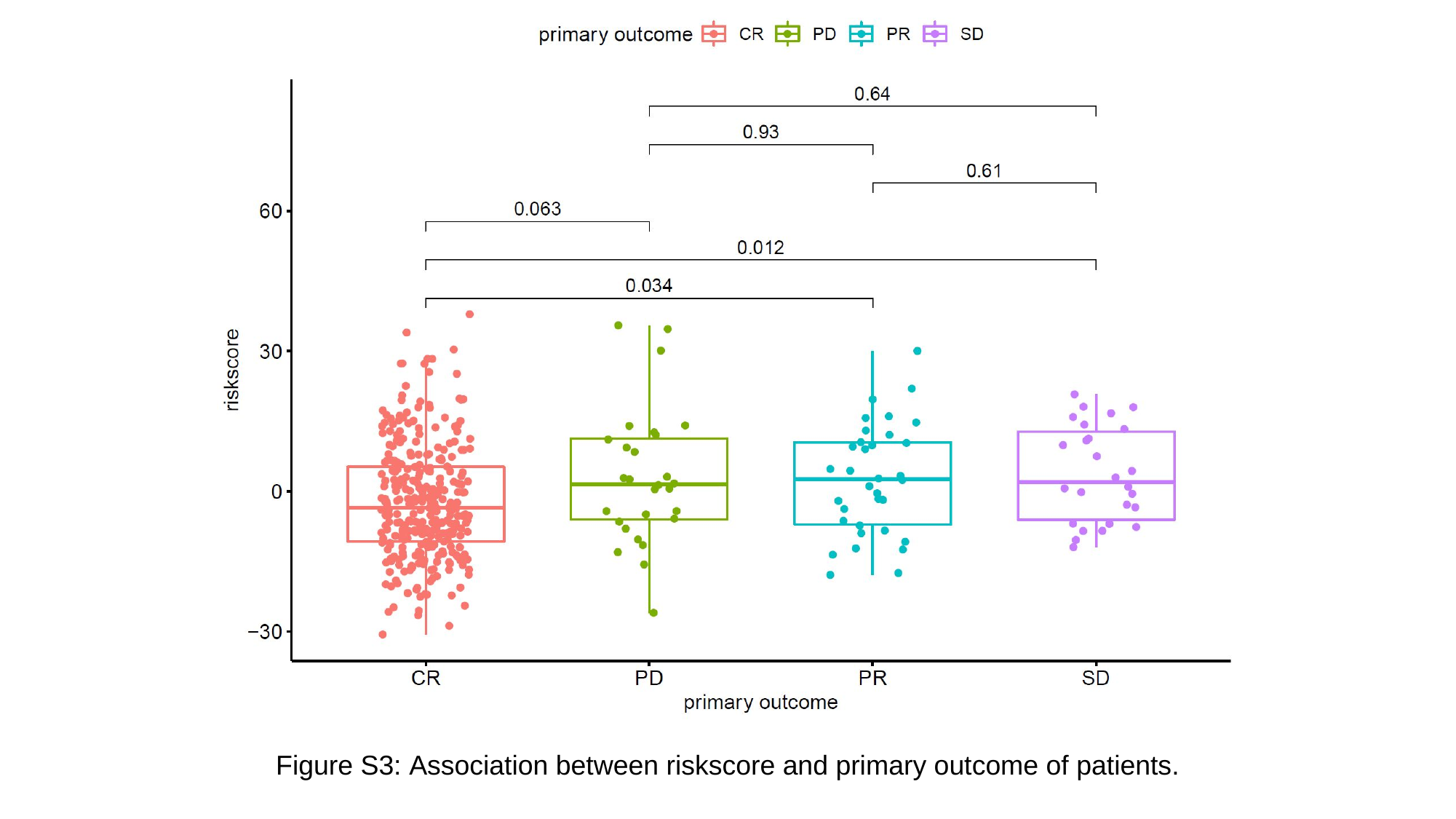

Figure S3: Association between riskscore and primary outcome of patients.

## Slide 4
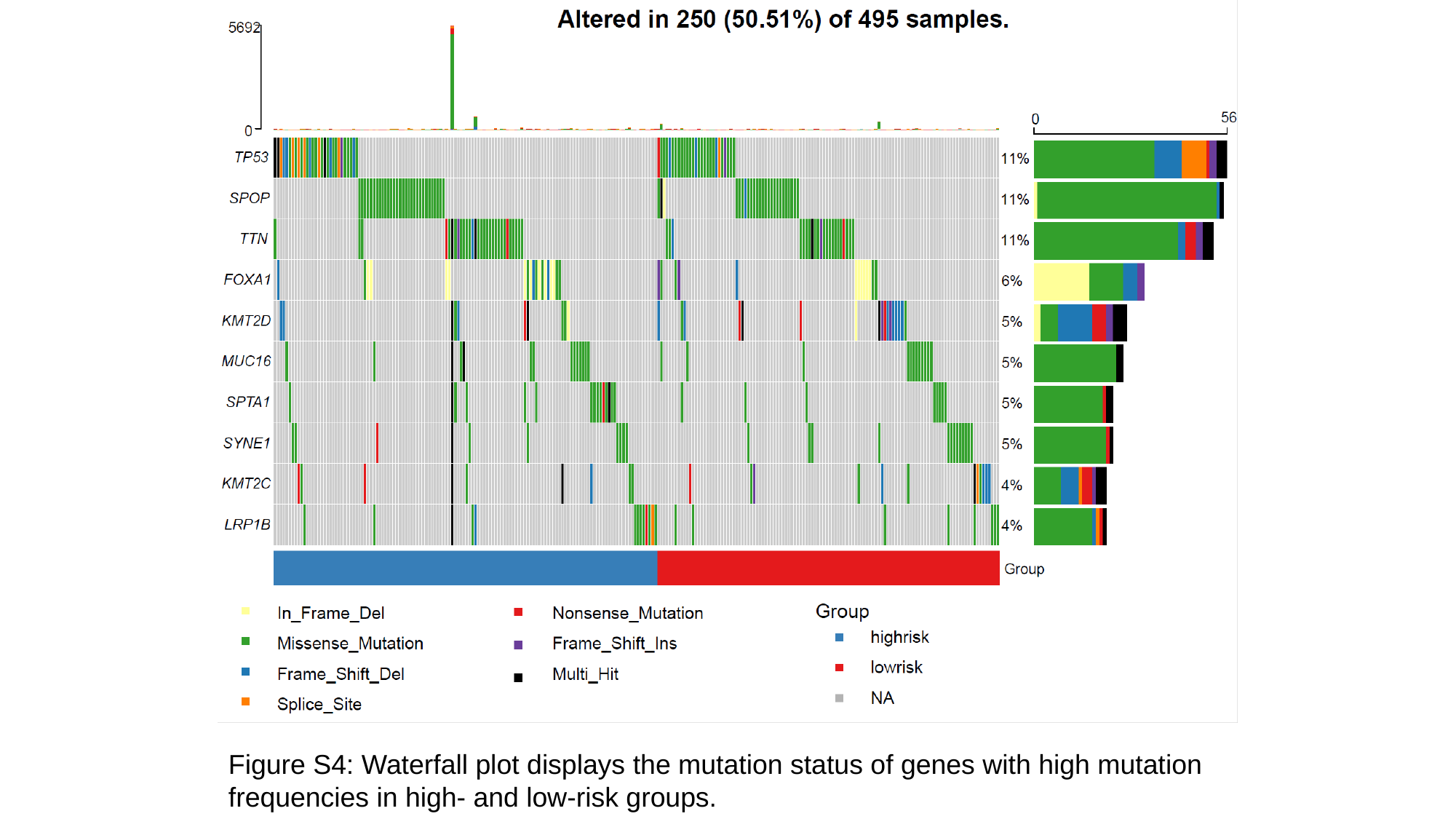

Figure S4: Waterfall plot displays the mutation status of genes with high mutation frequencies in high- and low-risk groups.

## Slide 5
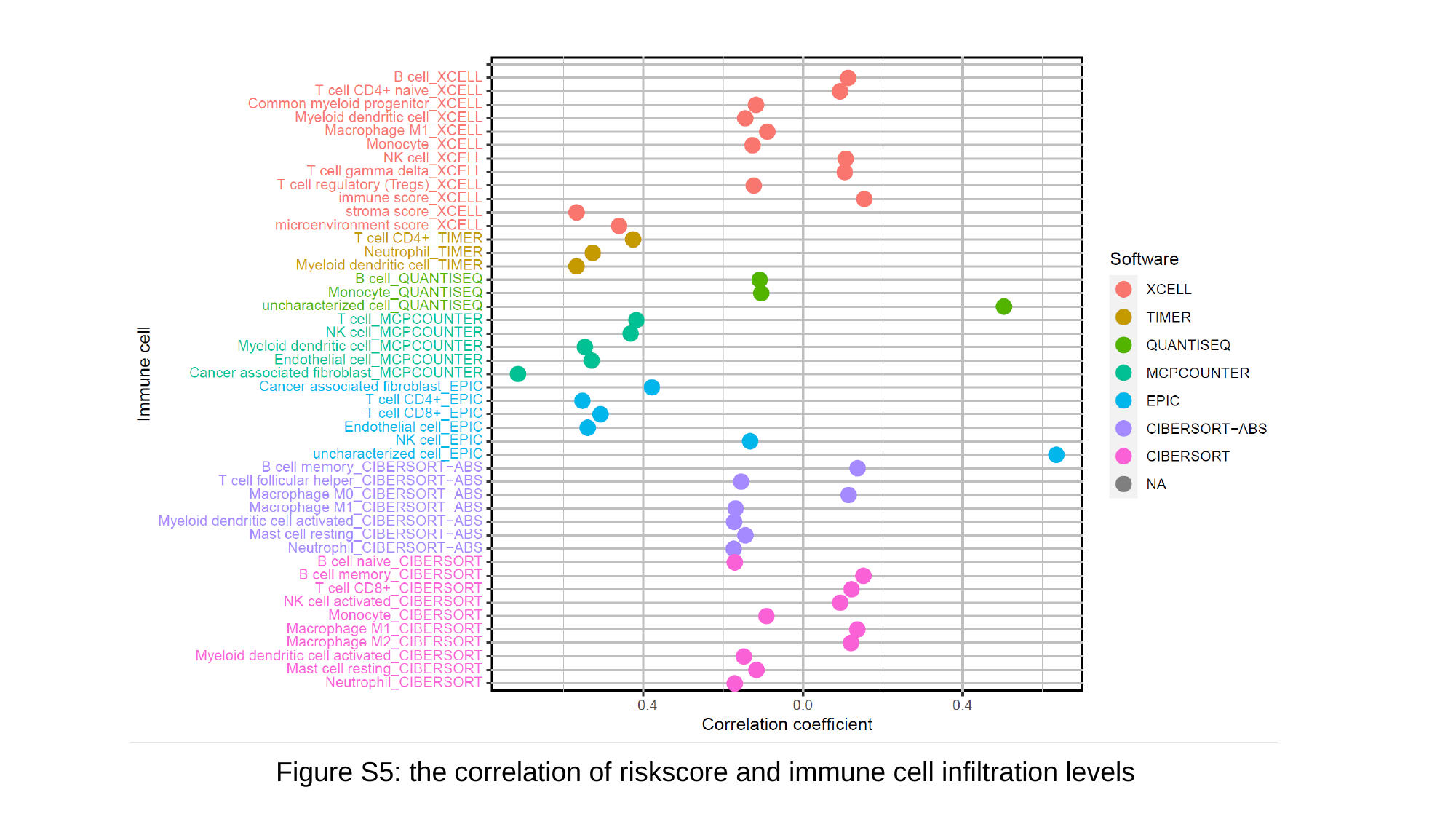

Figure S5: the correlation of riskscore and immune cell infiltration levels
