# Supplementary material for: Identification of a Gene Signature to Aid Treatment Decisions by Integrated Analysis of Mutated Genes Between Primary and Metastatic Prostate Cancer
Source: Front Genet. 2022 Apr 12;13:877086. doi: 10.3389/fgene.2022.877086 (PMC9041415; doi:10.3389/fgene.2022.877086)
Supplement: Supplementary file 4 [file Table2.DOCX]

**Abbreviations**

COSMIC: The Catalogue of Somatic Mutations in Cancer

ECM: Extracellular matrix

FC: Fold change

GDSC: Genomics of Drug Sensitivity in Cancer

GEO: Gene Expression Omnibus

GSVA: Gene set variation analysis

PCa: Prostate cancer

PCA: principal components analysis

ROC: Receiver operating characteristic

ssGSEA: Single sample gene set enrichment analysis

TCGA: The Cancer Genome Atlas

TMB: Tumor mutation burden

TME: Tumor microenvironment

**Website**

TCGA database: <https://portal.gdc.cancer.gov/>

GEO database: <https://www.ncbi.nlm.nih.gov/geo/>

COSMIC database: <https://cancer.sanger.ac.uk/cosmic>

MSigDB database: <https://www.gsea-msigdb.org/gsea/msigdb/>

GDSC database: <https://www.cancerrxgene.org/>
