# Supplementary material for: Identification of a Gene Signature to Aid Treatment Decisions by Integrated Analysis of Mutated Genes Between Primary and Metastatic Prostate Cancer
Source: Front Genet. 2022 Apr 12;13:877086. doi: 10.3389/fgene.2022.877086 (PMC9041415; doi:10.3389/fgene.2022.877086)
Supplement: Supplementary file 6 [file Table3.DOCX]

Table S1: The clinical characteristics of patients in the TCGA-PRAD cohort

| Characteristic | levels | Overall |
| --- | --- | --- |
| n |  | 499 |
| Age, n (%) | <=60 | 224 (44.9%) |
|  | >60 | 275 (55.1%) |
| Race, n (%) | Asian | 12 (2.5%) |
|  | Black or African American | 57 (11.8%) |
|  | White | 415 (85.7%) |
| T stage, n (%) | T2 | 189 (38.4%) |
|  | T3 | 292 (59.3%) |
|  | T4 | 11 (2.2%) |
| N stage, n (%) | N0 | 347 (81.5%) |
|  | N1 | 79 (18.5%) |
| M stage, n (%) | M0 | 455 (99.3%) |
|  | M1 | 3 (0.7%) |
| Gleason score, n (%) | 6 | 46 (9.2%) |
|  | 7 | 247 (49.5%) |
|  | 8 | 64 (12.8%) |
|  | 9 | 138 (27.7%) |
|  | 10 | 4 (0.8%) |
